# Optimization of Thermal Conductivity and Tensile Properties of High-Density Polyethylene by Addition of Expanded Graphite and Boron Nitride

**DOI:** 10.3390/polym15173645

**Published:** 2023-09-04

**Authors:** Lovro Travaš, Maja Rujnić Havstad, Ana Pilipović

**Affiliations:** Faculty of Mechanical Engineering and Naval Architecture, University of Zagreb, Ivana Lucica 5, 10000 Zagreb, Croatia; mrujnic@fsb.hr (M.R.H.); apilipovic@fsb.hr (A.P.)

**Keywords:** boron nitride, composite, expanded graphite, HDPE pipe, tensile properties, thermal conductivity

## Abstract

Due to its mechanical, rheological, and chemical properties, high-density polyethylene (HDPE) is commonly used as a material for producing the pipes for transport of various media. Low thermal conductivity (0.4 W/mK) narrows down the usage of HDPE in the heat exchanger systems. The main goal of the work is to reduce the vertical depth of the HDPE pipe buried in the borehole by increasing the thermal conductivity of the material. This property can be improved by adding certain additives to the pure HDPE matrix. Composites made of HDPE with metallic and non-metallic additives show increased thermal conductivity several times compared to the thermal conductivity of pure HDPE. Those additives affect the mechanical properties too, by enhancing or degrading them. In this research, the thermal conductivity and tensile properties of composite made of HDPE matrix and two types of additives, expanded graphite (EG) and boron nitride (BN), were tested. Micro-sized particles of EG and two different sizes of BN particles, micro and nano, were used to produce composite. The objective behind utilizing composite materials featuring dual additives is twofold: firstly, to enhance thermal properties, and secondly, to improve mechanical properties when compared with the pure HDPE. As anticipated, the thermal conductivity of the composites exhibited an eightfold rise in comparison to the pure HDPE. The tensile modulus experienced augmentation across all variations of additive ratios within the composites, albeit with a marginal reduction in tensile strength. This implies that the composite retains a value similar to pure HDPE in terms of tensile strength. Apart from the enhancement observed in all the aforementioned properties, the most significant downside of these composites pertains to their strain at yield, which experienced a reduction, declining from the initial 8.5% found in pure HDPE to a range spanning from 6.6% to 1.8%, dependent upon the specific additive ratios and the size of the BN particles.

## 1. Introduction

Nanocomposites are composites made of particles up to 100 nm large, while microcomposites contain particles sized from 0.1 to 100 µm. Generally, there is a significant effect on the mechanical and rheological properties of the composite caused by micro- and nano-constituents compared to the matrix material. Nanocomposites based on polymer matrix and non-polymer additives are the subject of various studies aiming for the enhancement of electric conductivity, antistatic features, tensile strength, flexural strength, water absorption, abrasion resistivity, etc. [1,2,3,4,5,6]. Due to the significantly higher thermal conductivity (*λ*) of carbon-based additives compared to polymers, the presence of these additives results in the increase of thermal conductivity of carbon–polymer composites [7]. The enhanced thermal properties of graphite are related to its structure, in which atoms of carbon build a hexagonal one-layered structure. Expanded graphite (EG) is one of the many modifications of graphite, which is produced by intercalation and can be exfoliated several hundred times compared to the original volume when exposed to heat. Three-dimensional wormlike structures of EG are the basis for achieving increased values of electro and thermal conductivity in EG–polymer composite [8]. Compared to polymers, most metals have a hundred times higher *λ* values. Some metal additives used in polymer matrices, such as nickel–copper alloy and titanium, are characterized by excellent chemical resistivity and can therefore be used as heat exchangers in media such as sea water or chemicals. On the other hand, polymer processing demands significantly lower temperatures (<300 °C) compared to metal processing [9]. Polyethylene, especially high-density polyethylene (HDPE), due to its low price, recyclability, nontoxicity, corrosion resistivity, and good processing properties, has a wide range of applications. Features of composites with HDPE as matrix are based on the interphase compatibility between matrix and additive, polarity between the contact surfaces of the matrix and additive, etc. The dispersion of additives in the matrix depends on size, shape, dispersion technique, equipment, and on processing parameters (time, temperature, etc.). To increase the dispersion of additive in matrix, and thus reduce the surface tension between components, various methods are applied. Some of them include the addition of maleic anhydride (MAH), resulting in an increase of strength, toughness, and ductility. Other methods include high speed shearing during the mixing of components (such as poly(methyl-methacrylate) and EG), resulting in similar improvements compared to MAH [10].

### 1.1. Composites with HDPE and EG

Sobaliček et al. exposed HDPE and untreated EG mixture to a temperature above the melting point of HDPE, which resulted in multiple times higher value of thermal conductivity compared to HDPE [11]. EG treated with poly(vinyl-alcohol) shows an increase of thermal conductivity in the polymer, even at low percentages of additives, which was researched by Yin et al. [12]. According to Panagiotis et al., the polystyrene (PS) matrix, compared to the HDPE matrix with EG additive, results in a multiple-fold increase in thermal conductivity [13]. A similar procedure and parameters were applied (temperature of kneader chamber of 185 °C, at 60 rpm/min, and for 10 min) for mixing HDPE/EG/carbon nano tubes (CNT) composite, with EG up to 20 wt.% and CNT up to 3 wt.%. It was then pressed at a temperature of 185 °C and pressure of 10 MPa. For the highest concentration of both additives, thermal conductivity reached a value of ~ 3 W/mK [14]. In their research, Sanchez et al. produced a composite with an ultra-high molecular weight polyethylene (UHMWPE) matrix and graphite as an additive by ultrasonic injection molding. Even at low mass percentage (7 wt.%), the tensile modulus of the composite increased 96% when compared to the tensile modulus of the matrix [15]. Abdelrazeq et al. researched the properties of a phase-change material made of HDPE matrix, paraffin wax, and 15 wt.% EG. The composite was exposed to UV radiation, temperature, and moisture. The highest value of thermal conductivity, 1.64 W/mK, was measured at the highest mass percentage of EG [16].

### 1.2. Composites with HDPE and BN

Muratov et al. used HDPE and hexagonal BN (hBN) with two particle sizes, 10 μm in mass proportions of 25% and 50% and 150 nm in 25 wt.%. The highest value of thermal conductivity, 2.08 W/mK, was achieved in the composite with 50 wt.% micro-sized hBN particles and without a compatibilizer. The yield strength for all composites reached between 22 MPa and 24.9 MPa, while the highest tensile modulus, 3829 MPa, was measured for the composite with 50 wt.% mass percentages of hBN micro particles and compatibilizer based on titanate (KR TTS) [17]. A composite with LDPE matrix and hexagonal boron nitride nanosheets (hBNNs) was studied by Ali et al., where hBNNs were added in a volume percentage up to 30%. A value of thermal conductivity of 1.46 W/mK was reached for the highest volume proportion of additive. The elasticity modulus reached 2.2 GPa for 25 vol.% and the tensile strength was 18.7 MPa for the same volume percentage of the additive [18]. A layered structure of LDPE/HDPE and BN composite was researched by Shang et al. BN was added in composites in 15 wt.%, which resulted in a measured thermal conductivity of 3.54 W/mK for the layered structure and 3.13 W/mK for the randomly oriented composite with the same mass percentage of BN [19]. A significant increase in thermal conductivity was achieved by Zhang et al. by stretching composite foil with HDPE matrix and boron nitride nanoplates (BNNPs) as additives. The measured thermal conductivity reached 3.1 W/mK for unstretched foil with 15 wt.% BNNP, while for stretched foil (with stretching ratio ***Λ*** = 5), thermal conductivity reached 106.2 W/mK [20]. A recycled PE matrix with high ratio of aluminum oxide and zinc oxide as matrix and BN as additive was used by Rasul et al. to produce the composite, where silane was used as a compatibilizer. The thermal conductivity of the matrix was 0.72 W/mK; for the composite with 5 wt.% of BN, it increased to 0.84 W/mK and for the composite with 5 wt.% of BN and 3 wt.% of silane, it reached 0.96 W/mK [21]. An increase of a composite’s thermal conductivity was achieved by Shi et al. by solid-state extrusion. Using UHMWPE as the matrix and BN as an additive with 50 wt.%, it reached a thermal conductivity of 23.03 W/mK [22]. In his research, Lebedev compared the thermal conductivity of two composites produced by injection molding. The thermal conductivity of the composite made of polyacrylic acid (PLA) and BN reached 0.67 W/mK, while the composite made of LDPE and BN reached a thermal conductivity of 0.7 W/mK for the same BN ratio of 40 wt.% [23]. Güzdemir et al. researched the increase of thermal conductivity for LDPE/BN composite in the shape of extruded film. The initial thermal conductivity of the LDPE matrix of 0.4 W/mK increased to 1.8 W/mK for the composite with 30 vol% of BN additive [24]. Other research, such as the study conducted by Yang et al., included a composite with HDPE matrix with BN (25 wt.%) and coconut shell carbon (3 wt.%), reaching a thermal conductivity of 0.943 W/mK [25].

Considering the superior thermal conductivity inherent in both BN and EG compared to PE, it is foreseeable that the EG/BN/HDPE composite will exhibit significantly higher thermal conductivity in comparison to HDPE. While this particular composite has not yet been explored, the hypothesis suggests a substantial multiple-fold increase in thermal conductivity. The potential application of such a material would primarily target heat exchangers, an area where mechanical properties carry equal significance alongside thermal attributes. Consequently, the composite material will undergo testing to assess its tensile properties, providing valuable insights into its suitability for this purpose.

While existing research predominantly investigated either EG/PE or BN/PE composites with regards to mechanical properties, there is a lack of examination of their synergistic effects. According to the literature of previous authors, it is necessary to add up to 50% EG and 50% BN individually. The point of this work is to reduce the percentages of EG and BN and to obtain better thermal conductivity and tensile properties. Therefore, it is necessary to see how both additives together affect the properties. Additionally, the study investigated the interaction between the particle size of boron nitride and the incorporation of expanded graphite within the HDPE matrix. Based on a literature review, it is anticipated that BN will exert a more pronounced influence on tensile properties compared to EG. Furthermore, the work optimized the results according to the input and output parameters for the actual application of that material.

## 2. Materials and Methods

### 2.1. Materials

HDPE 6060R (*Sabic*, Riyadh; Saudi Arabia) was chosen as a matrix material in a granulate form. The type of material is PE-100, with granulate density of 0.959 g/cm^3^ and melting point at 124 °C. HDPE was chosen because of its easy processability, low cost, light weight, and low melting point. This material is a classic material for the production of pipes to be installed in a borehole for the transfer of media. SABIC^®^ Vestolen A 6060R 10000 (black) is a grade which has a high density and a bimodal distribution of the molecular mass. Due to its profile of properties, this material is typically used for gas, drinking water, and wastewater piping. This material meets (inter)national standards for use in gas, drinking water, and wastewater piping MRS class ISO 12162 MPa = 10.0 (PE 100). Expanded graphite *Sigratherm GFG75* was acquired from the producer *SGL Carbon* (Austria) in a powdered form. It has a density of 2.25 g/cm^3^, with an average particle size of 75 µm. The manufacturer specifies > 98% carbon in the material. Boron nitride with micro-sized particles (35 µm) named *HeBoFill* from *Henze* (Lauben; Germany) has a density of 2.25 g/cm^3^, while boron nitride with nano-sized particles (70 nm) produced by *IoLiTec* (Heilbronn; Germany) has a density of 2.3 g/cm^3^. Both micro- and nano-sized BN additives have a hexagonal crystal structure. Compatibilizer PE-g-MAH was acquired from *BOC Sciences* and was added into each composite in 5 wt.%. All additional information can be seen in the data sheets from the manufacturer. The designations for the composite with expanded graphite and micro-particles of BN are HDPE/EG/mBN, and with expanded graphite and nanoparticles of BN HDPE/EG/nBN.

### 2.2. Processing

A laboratory twin screw kneader (*MetaStation 4E*, manufacturer *Brabender*; Duisburg; Germany) was used for mixing the components. A chamber sized 50 cm^3^ was heated up to 200 °C, with a rotation speed of 60 min^−1^, and for a duration of 20 min. Firstly, HDPE was added into the mixing chamber, followed by the compatibilizer, and finally EG and BN. After mixing was done, the composite was cooled down to room temperature and milled in a mechanical mill (*SM100*, manufacturer *Retsch*; Haan; Germany) into average particles sizes of 4 mm. The size of the particles is controlled by selecting a sieve that is replaceable. Ultimately, the composite was formed through compression molding, involving a hot pressing process at 15 MPa and 180 °C for a duration of 10 min to create the test specimens. Subsequently, the molded specimens were cooled to room temperature within a closed mold, utilizing water as the cooling medium. The composites are marked according to the mass percentage of additives, i.e., HDPE/EG5/mBN15 means that 5 wt.% of expanded graphite and 15 wt.% micro-sized particles of boron nitride were added into the HDPE matrix. Combinations of additive mass proportions in the matrix were determined according to the central composite design in the *Design Expert* software. Eleven experimental runs were determined for both micro- and nano-sized BN particles and are shown in Table 1 and Table 2. The thermal conductivity of composites was measured according to ISO 22007-2:2022 by the transient hot bridge method. The thermal conductivity was determined on the *Linesis THB Advanced* device. A THBN1 sensor was used for the measurement with a measurement error of 2%. The measurements were carried out at a current of 0.056 A and at a temperature of 23 °C, and the measurement time was 50 s. Tensile properties were tested on a universal testing machine *Shimadzu AGS-X* with a maximum force of 10 kN according to HRN EN ISO 527-2 at a speed of 1 mm/min. The test was carried out on type 1BA test specimens with dimension 110 × 10 × 2 mm. The measurement error on the universal testing machine is 0.5% in the measurement range from 10 to 10,000 N. For each composite, six specimens were produced by the described procedure for tensile properties and two for thermal properties. Thermal conductivity was measured at 10 places on the test specimens in contact. The mean value and standard deviation were calculated and are shown in Table 1 and Table 2. In the table, after the ± sign, the standard deviation, that is, the dispersion of data in the conducted testing of six test specimens, is shown. The effect of additives on the thermal and tensile properties of pure HDPE matrix was tested according to the design of the experiment generated by software *Design Expert,* using response surface methodology. Data acquired by testing were processed by ANOVA (analysis of variance) linear modeling method with three center points. Values crossed out in Table 1 and Table 2 are excluded from further data analysis because of a high result deviation from the center point in the model calculated by *Design Expert*. Along with the results for different ratios of EG and BN in the HDPE matrix, the results of pure HDPE are also presented. The maximum and minimum limits of the percentage of BN for the HDPE/EG/mBN and HDPE/EG/nBN composites are different because the point is to see if the same or similar properties can be obtained with a smaller amount of nano particles of BN as with the use of cheaper micro particles of BN. Furthermore, only one particle size was chosen for the EG additive because the selected material proved to be the best with preliminary experiments. In the table, crossed values refer to values that were excluded from further analysis because they deviate from the model.

## 3. Results

Composites were divided into two groups, one with nano-sized particles of BN and the other with micro-sized particles of BN, to compare thermal and tensile properties depending on additive proportions.

### 3.1. Thermal Properties

Even at the lowest proportions, the inclusion of additives led to an increase in the thermal conductivity of the composites, reaching its peak value at the greatest mass proportions of additives. From a starting value of 0.37 W/mK for pure HDPE, the thermal conductivity increased up to 3.00 W/mK for the HDPE/EG22/mBN22 composite and to 2.09 W/mK for the HDPE/EG22/nBN6.5 composite. In percentages, this means an increase of 710% and 465%, respectively, compared to the thermal conductivity of HDPE. For the same proportion of 15 wt.% of micro or nano BN additive, the value of the thermal conductivity is greater for mBN (2.13 W/mK), while for the same mass percentage of nBN, the thermal conductivity is 1.95 W/mK. Higher thermal conductivity values for the HDPE/EG/mBN composite indicate that a better thermal pathway is achieved through larger particles. The contribution of additives to the thermal conductivity of composites is comparable for both EG and BN. This similarity can be attributed to the notable thermal conductivity inherent in both EG and BN, stemming from their hexagonal crystal lattice structure (Table 3 and Table 5).

ANOVA analysis of variance indicates that the 2-factor interaction (2FI) model best fits the influence of additives EG and mBN on the thermal conductivity. The details of analysis are shown in Table 3. In the table, df stands for degrees of freedom.

The model *F*-value (variation between sample means) of 17.43 implies that the model is significant. There is only a 0.44% chance that an *F*-value this large could occur due to noise. *p*-values less than 0.05 indicate that model terms are significant. In this case, both factor A and B (percentage of EG and mBN) are significant model terms. Values greater than 0.1 indicate that the model terms are not significant. If there are many insignificant model terms (not counting those required to support hierarchy), model reduction may improve the model. The lack of fit *F*-value of 0.6637 implies that the lack of fit is not significant relative to the pure error. There is a 64.76% chance that a lack of fit *F*-value this large could occur due to noise. Non-significant lack of fit is good because it means that the model fits.

The statistical data (mean value, standard deviation, and *R*^2^) about the model are given in Table 4. The coefficient of determination *R*^2^ is a measure of deviation from the arithmetic mean which is explained by the model. The closer *R*^2^ is to 1, the better the model follows the data, that is, the phenomenon is better explained.

From Table 4, it can be concluded that the model followed the data very well since the coefficient of determination is *R*^2^ = 0.9127. The predicted *R*^2^ of 0.683 is in reasonable agreement with the adjusted *R*^2^ of 0.8604; i.e., the difference is less than 0.2. An adequate precision measures the signal to noise ratio. A ratio greater than 4 is desirable. The ratio of 12.805 indicates an adequate signal.

Predicted thermal conductivity *λ* for HDPE/EG/mBN composite can be described by Equation (1) in actual parameters:

*λ* = 0.582 + 0.043 × EG + 0.029 × mBN + 0.002 × EG × mBN (1)

where: *λ* (W/mK)—thermal conductivity, EG and mBN are the mass percentages of expanded graphite and micro boron nitride in %.

In the case of the HDPE/EG/nBN composite with nano BN, ANOVA analysis implies that the linear model best describes the influence of additives on thermal conductivity. Analysis of variance is shown in Table 5.

The model *F*-value (variation between sample means) of 16.82 implies that the model is significant. There is only a 0.35 % chance that an *F*-value this large could occur due to noise. P-values less than 0.05 indicate that the model terms are significant. Both Factor A and B (EG and nBN additives) are significant model terms. Lack of fit *F*-value of 0.44 implies that the lack of fit is not significant relative to the pure error. There is a 78.33% chance that a lack of fit *F*-value this large could occur due to noise.

Table 6 shows basic statistical data about the model.

The predicted *R*^2^ of 0.7249 is in reasonable agreement with the adjusted *R*^2^ of 0.7982. The ratio of 10.0348 indicates an adequate signal. From Table 6, it can be concluded that the model followed the data very well since the coefficient of determination is *R*^2^ = 0.8486.

The predicted thermal conductivity *λ* for HDPE/EG/nBN composite can be described by Equation (2) in actual parameters:

*λ* = 0.210348 + 0.068865 × EG + 0.047779 × nBN (2)

where: *λ* (W/mK)—thermal conductivity, EG and nBN are the mass percentages of expanded graphite and nano boron nitride in %.

**Figure 1:**
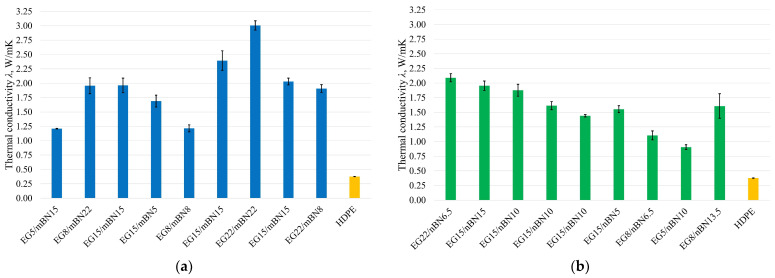
Thermal conductivity of all tests run: (**a**) HDPE/EG/mBN composites; (**b**) HDPE/EG/nBN composites.

**Figure 2:**
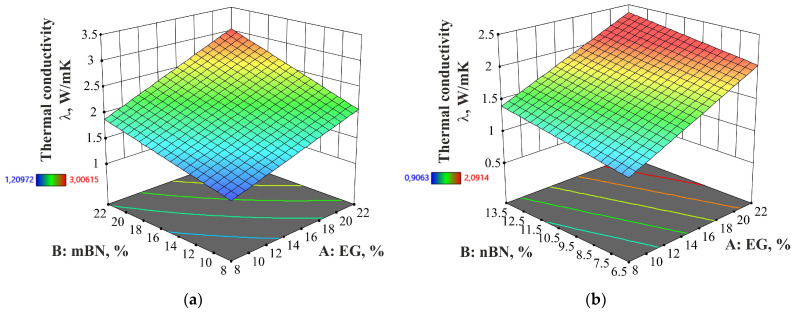
Dependence of proportion of EG and BN additives on the thermal conductivity of: (**a**) HDPE/EG/mBN composite; (**b**) HDPE/EG/nBN composite.

The contribution of both additives in HDPE/EG/mBN and HDPE/EG/nBN composites proves the assumption that the structure of these materials will lead to an increase of thermal conductivity of the composites. Two-factor interaction and a linear model that describe the contribution of additives for EG/mBN and EG/nBN can be applied to predict thermal conductivity *λ* accurately. Figure 1 shows the thermal conductivity for each run of the experiment (except for those excluded from the analysis, in this case, run 4 and 8 for composites HDPE/EG/mBN and run 1 and 9 for composites HDPE/EG/nBN) and pure HDPE to compare the obtained results, while Figure 2 shows the thermal conductivity dependence on the mass proportions of additives. In addition to the values, error bars, i.e., deviations from the results, have been added to the diagram. The diagram indicates a significant impact of boron nitride particle size on thermal conductivity. Notably, the addition of BN nanoparticles to pure HDPE results in a 1 W/mK lower thermal conductivity value than with larger BN particles. It can be concluded that larger BN particles will yield a more substantial enhancement in thermal conductivity.

### 3.2. Tensile Properties

#### 3.2.1. Tensile Strength

The highest value of tensile strength for the composite with micro-particles reached 24.37 MPa (EG15/mBN25) and 24.03 MPa for the composite with nano-particles of BN (EG15/nBN15). For both groups of composites (micro- and nano-sized BN particles), this was the highest ratio of BN. Accordingly, the lowest values of tensile strength were achieved for EG5/mBN15 and EG15/nBN5. With various percentages of both additives, the approximate tensile strength value of the pure HDPE was maintained (25.58 MPa).

Based on the analysis of variance (ANOVA), the linear model provides the most suitable fit for assessing the impact of additives on the tensile strength of the composite containing micro-sized BN. Analysis of variance is shown in Table 7.

The model *F*-value (variation between sample means) of 9.78 implies that the model is significant. There is only a 0.71% chance that an *F*-value this large could occur due to noise. In this case, factor A–EG is a significant model term. A lack of fit *F*-value of 0.32 implies that the lack of fit is not significant relative to the pure error. There is an 88.01% chance that a lack of fit *F*-value this large could occur due to noise.

Furthermore, statistical data about the model for tensile strength is shown in Table 8.

The predicted *R*^2^ of 0.4942 is in reasonable agreement with the adjusted *R*^2^ of 0.6371; i.e., the difference is less than 0.2. An adequate precision ratio of 7.857 indicates an adequate signal.

The tensile strength *σ*_m_ for HDPE/EG/mBN composite can be described by Equation (3):

*σ*_m_ = 16.5691 + 0.2393 × EG + 0.0997 × mBN (3)

where: *σ*_m_ (N/mm^2^ or MPa)—tensile strength, EG and mBN are the mass percentages of expanded graphite and micro boron nitride in %.

As for composites HDPE/EG/nBN, ANOVA implies that the linear model best describes the influence of additives on tensile strength. Analysis of variance is shown in Table 9.

In this case, both additives EG and nBN are significant model parameters. The lack of fit *F*-value of 0.35 implies that the lack of fit is not significant relative to the pure error. There is an 86.44% chance that a lack of lit *F*-value this large could occur due to noise, which indicates a well-chosen model for tensile strength.

The statistical data about model for tensile strength for HDPE/EG/nBN composite are shown in Table 10.

The predicted *R*^2^ of 0.4641 is in reasonable agreement with the adjusted *R*^2^ of 0.6214. The adequate precision ratio of 8.1705 indicates an adequate signal. *R*^2^ is only 0.6971, which is a slightly lower value, but in accordance with the other presented parameters of the model, both additives are significant for the display of tensile strength. When comparing the *R*^2^ values for the HDPE/EG composite with micro- or nano-sized BN particles, the values are the same.

The tensile strength *σ*_m_ for HDPE/EG/nBN composite can be described by Equation (4):

*σ*_m_ = 18.80401 + 0.096851 × EG + 0.206639 × nBN (4)

where: *σ*_m_ (N/mm^2^ or MPa)—tensile strength, EG and nBN are the mass percentages of expanded graphite and nano boron nitride in %.

**Figure 3:**
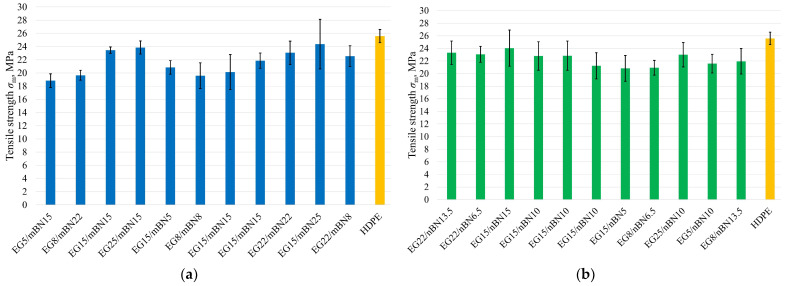
Tensile strength of all tests run: (**a**) HDPE/EG/mBN composites; (**b**) HDPE/EG/nBN composites.

**Figure 4:**
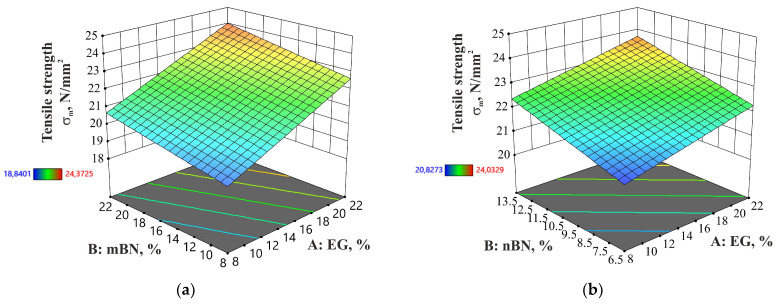
Dependence of percentage of EG and BN additives on the tensile strength of: (**a**) HDPE/EG/mBN; (**b**) HDPE/EG/nBN composite.

In the case of the HDPE/EG/nBN composite, both additives exhibit a comparable effect on tensile strength. However, in the HDPE/EG/mBN composite, it is evident that EG holds a greater influence over the tensile strength. Figure 3 displays tensile strength for each run of the experiment, while Figure 4 shows the dependence of tensile strength on the amount of EG and BN.

#### 3.2.2. Tensile Modulus

In addition to tensile strength, the values of the tensile modulus for both composites are dispersed in the range from 1072 MPa (HDPE/EG5/mBN15) to 2153 MPa (HDPE/EG15/mBN25) and from 1144 MPa (HDPE/EG5/nBN10) to 2006 MPa (HDPE/EG25/nBN10). In contrast to the pure HDPE value of 1104 MPa, the highest value observed for EG15/mBN25 showcased a 95% increase. As expected, the values of the tensile modulus increased with the addition of additives to the composite. For HDPE/EG/mBN, the ANOVA implies that the reduced quadratic model fits best to describe the impact of additives on tensile modulus, as shown in Table 11.

Both factor A and B (EG and mBN) are significant model terms (p-value of 0.0001). The value of 0.48 implies that the lack of fit *F*-value is not significant relative to pure error—there is a 73.14% chance that a lack of fit *F*-value this large could occur due to noise, which indicates that the model is well chosen.

The statistical data about the model for tensile modulus for HDPE/EG/mBN composite are shown in Table 12.

From Table 12, it can be concluded that the model followed the data very well since the coefficient of determination is *R*^2^ = 0.975.

The tensile modulus *E* for HDPE/EG/mBN composite can be described by Equation (5):

*E* = −493.85333 + 117.1943 × EG + 74.78531 × mBN − 1.91297 × EG × mBN − 1.44711 × EG^2^ (5)

where: *E* (MPa)—tensile modulus, EG and mBN are the mass percentages of expanded graphite and micro boron nitride in %.

Based on the analysis of variance, the most suitable model to describe the interaction of additives with the tensile modulus of the HDPE/EG/nBN composite is the two-factor interaction model. The analysis of measured values is shown in Table 13.

In this case, only factor A (EG) is a significant model term (*p*-value of 0.0007). The value of 0.35 implies that the lack of fit *F*-value is not significant relative to pure error—there is an 83.32% chance that a lack of fit *F*-value this large could occur due to noise.

The statistical data about the model for tensile modulus for HDPE/EG/nBN composite are shown in Table 14.

*R*^2^ in the case of HDPE/EG/mBN is closer to the number one than in the case of the application of nano particles boron nitride in composite, but the value of 0.875 and the values of other statistical data indicate that the model is well chosen.

The tensile modulus *E* for HDPE/EG/nBN composite can be described by Equation (6):

*E* = 658.69725 + 56.78545 × EG + 32.88882 × nBN − 1.42449 × EG × nBN (6)

where: *E* (MPa)—tensile modulus, EG and nBN are the mass percentages of expanded graphite and nano boron nitride in %.

**Figure 5:**
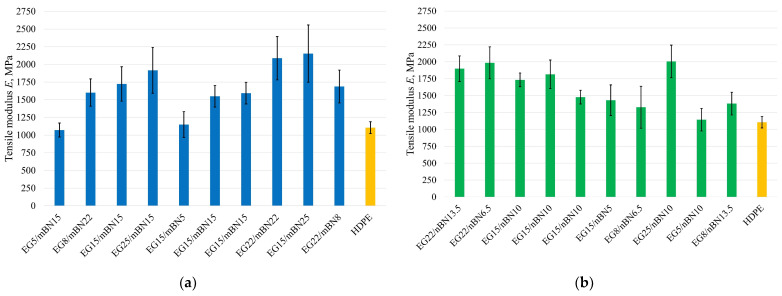
Tensile modulus of all tests run: (**a**) HDPE/EG/mBN composites; (**b**) HDPE/EG/nBN composites.

**Figure 6:**
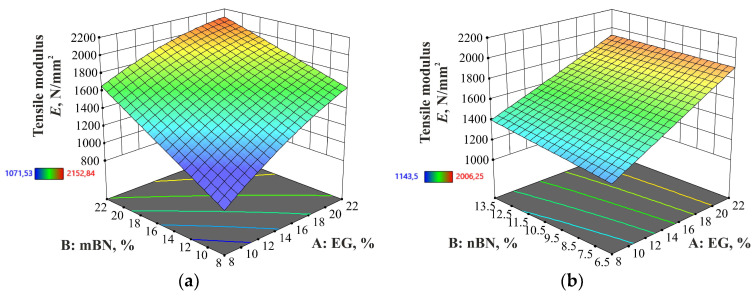
Dependence of percentage of EG and BN additives on the tensile modulus of: (**a**) HDPE/EG/mBN; (**b**) HDPE/EG/nBN composite.

In the case of the HDPE/EG/mBN composite, both additives contribute to the impact on the tensile modulus. Conversely, in the HDPE/EG/nBN composite, only EG demonstrates an influential effect. Figure 5 illustrates the tensile modulus for each experimental run and pure HDPE, whereas Figure 6 illustrates the relationship between the mass proportion of additives and the tensile modulus.

#### 3.2.3. Strain at Yield

Table 15 gives the ANOVA results for the strain at yield for HDPE/EG/mBN composites and Table 16 shows the basic statistical data for the model.

Table 15 reveals that both factors A and B (EG and mBN) significantly impact the strain at yield. In contrast, the lack of fit is not statistically significant, suggesting that the selected linear model adequately followed the strain at yield data.

From Table 16, it can be concluded that the model followed the data very well since the coefficient of determination is *R*^2^ = 0.8103.

The strain at yield *ε*_y_ for HDPE/EG/mBN composite can be described by Equation (7):

*ε*_y_ = 7.89820 − 0.117499 × EG − 0.158257 × mBN (7)

where: *ε*_y_ (%)—strain at yield, EG and mBN are the mass ratio of expanded graphite and micro boron nitride in %.

Table 17 gives the analysis results for the strain at yield for HDPE/EG/nBN composites and Table 18 shows the basic statistical data for the model. Table 17 indicates that factors A, B, and A^2^ exert a significant influence on the strain at yield of HDPE/EG/nBN composites.

From the given data in Table 18, it can be concluded that the model followed the data excellently. The coefficient of determination is *R*^2^ = 0.9983, which is higher than the determination coefficient for the composite HDPE/EG/mBN. This means that the presented quadratic model follows the data very well.

The strain at yield *ε*_y_ for HDPE/EG/nBN composite can be described by Equation (8):

*ε*_y_ = 8.50832 − 0.269906 × EG − 0.125754 × nBN − 0.001098 × EG × nBN + 0.002676 × EG^2^ + 0.002979 × nBN^2^ (8)

where: *ε*_y_ (%)—strain at yield, EG and nBN are the mass percentages of expanded graphite and nano boron nitride in %.

**Figure 7:**
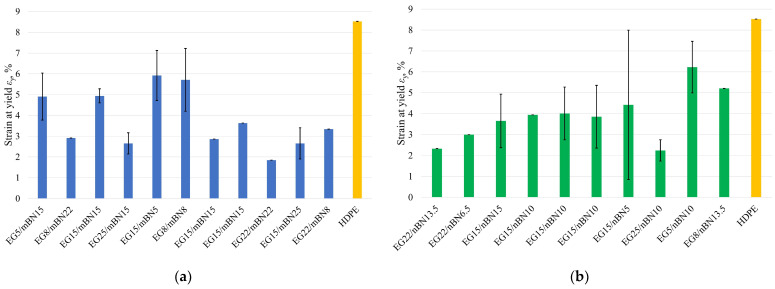
Strain at yield of all tests run: (**a**) HDPE/EG/mBN composites; (**b**) HDPE/EG/nBN composites.

**Figure 8:**
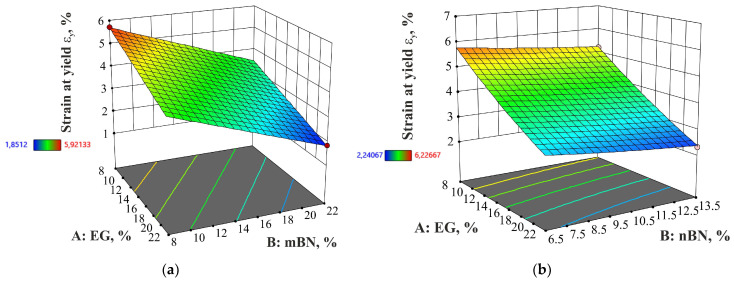
Dependence of percentage of EG and BN additives on the strain at yield of: (**a**) HDPE/EG/mBN; (**b**) HDPE/EG/nBN composite.

Figure 7 displays the strain at yield for each run of the experiment and pure HDPE, while the dependence of the mass percentage of additives on the strain at yield is shown in Figure 8.

### 3.3. Optimization

Software package *Design Expert* also includes an optimization module, in which optimization can be performed based on the desirability functions. The optimization process searches for a combination of factor values that simultaneously satisfy the criteria (wishes and priorities) placed on each of the responses and factors.

In this research, the optimization criteria (for optimal solution) were maximum thermal conductivity and strain at yield, while the tensile modulus and tensile strength with input percentage of EG and BN were within the chosen limits of the experiment.

The optimization was carried out in accordance with the basic goal of achieving the highest possible thermal conductivity within the selected limits of the input data (percentage of EG and BN additives in the HDPE matrix). Regarding the tensile properties, given that the tensile strength results for all combinations of percentages of additives in the HDPE matrix approximately maintained the value of pure HDPE, and the modulus in all combinations is greater than pure HDPE regardless of the size of the boron nitride particles, the values of the entire obtained spectrum results were considered for optimization. However, for easier processing, the strain at yield value should be as high as possible. Such a combination of the selected values for optimization of HDPE/EG/mBN composite gives the result presented in Table 19.

For the given optimization conditions, eleven solutions were found with desirability *d* = 0.449, which did not completely satisfy the set criteria/goal (thermal conductivity is only 1.9 W/mK). Such a low desirability is the consequence of trying to achieve the highest value of strain at yield. However, when this value is constrained to fall within the obtained limits, the desirability rises to *d* = 0.727.

The optimization constraints and criteria for version 2 for HDPE/EG/mBN composite are given in Table 20.

**Figure 9:**
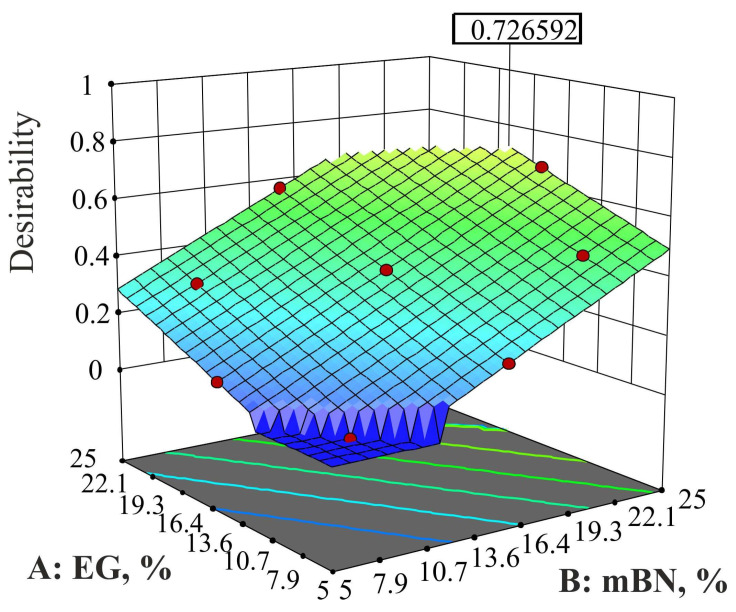
Desirability curve for the solution of HDPE/EG/mBN composite according to Table 18.

The desirability curve for the percentages of EG and mBN within the optimization constraints is shown in Figure 9.

The optimization of HDPE/EG/nBN composite gives the results presented in Table 21.

**Figure 10:**
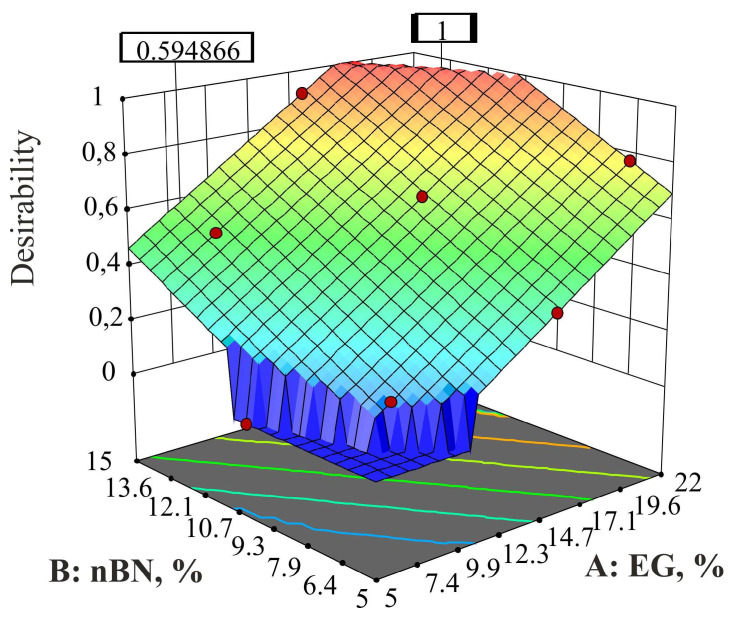
Desirability curve for the solution of HDPE/EG/nBN composite according to Table 18.

Under the specified optimization conditions for the HDPE/EG/nBN composite, the desirability stands at *d* = 0.655. This is unquestionably an improvement compared to the scenario involving composites with micro-sized BN particles. However, by adhering to the same assumption, it becomes feasible to elevate the desirability to its highest value of *d* = 1 (as shown in Figure 10 and Table 22).

The desirability curve for the percentages of EG and mBN within the optimization constraints is shown in Figure 10.

## 4. Discussion

In comparison with research carried out by Sever et al., which included an EG/HDPE composite with up to 40 wt%. of EG, the achieved values of tensile strength are similar.

In line with the findings of Sever et al., the inclusion of an additive led to a rise in tensile strength, increasing from 26.93 MPa for HDPE to 31.97 MPa at a 40 wt.% content of EG [26]. Considering the starting value of HDPE 25.58 MPa, a tensile strength of 24.37 MPa for HDPE/EG15/mBN25 shows a slight decrease compared to the 18% increase measured by Sever et al. Moreover, it is worth noting that both this study and the research by Sever et al. employed an identical total additive proportion ratio of 40 wt.%. However, it is important to highlight that the study conducted by Sever et al. focused solely on a single component. Li et al. achieved a tensile strength of 26.90 MPa for EG additive of 50 wt.%, at a mixing temperature of 190 °C and for a duration of 30 min [27]. Soboličak et al. reported an increase in tensile modulus up to 1440 MPa at an EG of 50 wt.%, but also a decrease of tensile strength by 40% compared to pure HDPE [11], which is comparable to the tensile modulus results obtained for some combination of HDPE/EG/mBN and HDPE/EG/nBN composites. Zhang et al. blended BN and HDPE composite with the addition of a PE-g-MAH compatibilizer, achieving a tensile strength of ~28 MPa with a BN of 20 wt.% [28]. With the same percentage of BN, Zhang et al. reached the highest value of tensile strength (~28.5 MPa), while with the further addition of BN, the value of tensile strength decreased [29]. This stands in opposition to the findings presented in this study, as the introduction of any amount of additive percentage consistently resulted in a decrease in tensile strength. In the research mentioned above, the size of the EG particles was 5 µm, while the sizes of the BN particles were 4 µm and 5 µm [26,28,29].

Compared to [26], where a tensile modulus of 3390 MPa was achieved for 40 wt.% of EG, and [11], where the tensile modulus reached 1440 MPa at 50 wt.% of EG, in our tests, only 15% EG and 25% mBN were enough to achieve 2153 MPa, which is 1.5 times higher than in the research in [11], i.e., 1.6 times less than in the research in [26]. The question arises as to why, despite the substantial 50 wt.% proportion utilized in the investigation [11], a comparatively small tensile modulus value was attained. The tensile property values typically arise from the bonding facilitated by compatibilizers between the additive and matrix, resulting in improved interfacial connections among these three phases. The increase of tensile modulus, while retaining consistent tensile strength, indicates the superior mechanical properties of additives compared to the matrix, particularly as the mass proportion of additives increases. Sofiti and Berto have reported that the tensile modulus of EG as an individual separate constituent falls within the range of 0.5 GPa to 3 GPa, whereas a single crystal of graphite exhibits a modulus of 36.5 MPa [30]. Compared to EG, a much higher tensile modulus of BN monolayer structure was reported by Falin et al., 0.865 TPa, while the tensile strength of bilayer structured BN reported by Song et al. was 0.334 TPa [31,32]. The findings of Cheurasia et al. from an investigation into the mechanical characteristics of composites featuring a PE matrix and hBN as an additive revealed that, even at a modest wt.% of BN, there was a significant 64% enhancement in the tensile modulus and a 27% increase in the tensile strength of the composite containing 5 wt.%, in contrast to the pure HDPE material [33]. Lee et al. reported a rise in tensile modulus by 127% and an increase in yield strength by 69% when utilizing BNNP as a 5 wt.% additive, as compared to the pure PE [34]. While experiencing notable improvements in strength and modulus, the introduction of any proportion of additives to the HDPE matrix results in a reduction in the strain at yield.

Compared to our results, Wieme et al. reported a higher value of thermal conductivity for in-plane measurement (4.03 W/mK). For through plane conductivity, which was used in this research too, Wieme reported a thermal conductivity of 0.69 W/mK [7]. With a high percentage of EG (50 wt.%) in HDPE, Soboliček reported a thermal conductivity of 2.18 W/mK, which is an increment of 372% compared to the HDPE matrix [11]. Our tests reveal that the HDPE/EG22/mBN22 composite exhibits a thermal conductivity of 3 W/mK. This value showcases a significant 137% increase when compared to the study conducted by Soboliček et al., even when employing only half the quantity of EG. A slightly higher percentage of EG (55 wt.%) in the HDPE matrix resulted in an increase in the thermal conductivity of composites (1.97 W/mK), as evidenced in a study by Klonos et al. [13], which can be compared with HDPE/EG15/BN15 regardless of the size of BN particles. Research on composites with BN as an additive show similar thermal properties to EG at the same percentage. Muratov et al. reported an increase of thermal conductivity to 2.08 W/mK for the HDPE/BN composite with 50 wt.% of a micro-sized hexagonal BN additive. For the same additive ratio, which was treated with titanate, thermal conductivity reached 0.9 W/mK [17]. Better results were reported by Rasul et al., who applied silane as a coupling agent. With the inclusion of 5 wt.% BN nanosheets, the thermal conductivity rose to 0.96 W/mK, whereas the matrix itself exhibited a thermal conductivity of 0.72 W/mK. The extraordinarily high value of matrix thermal conductivity is explained by the high mass proportion of aluminum oxide and zinc oxide, totaling 37% [21]. The importance of a coupling agent was researched by Zhang et al., where BN was treated with PE-g-MAH. The difference between treated and non-treated additive was obvious, 2.6 W/mK compared to 2.2 W/mK, respectively [28]. The literature surveyed from various authors reveals considerable disparities in thermal conductivity outcomes. It is noteworthy that achieving a thermal conductivity of 3 W/mK often demands substantial quantities of expanded graphite. However, our experiments demonstrate that this value can be attained using just half the EG amount when incorporating a specific proportion of BN. Notably, the overall additive quantity required to achieve this desired thermal conductivity is smaller in comparison to other investigations.

Salavagione et al. [35], to increase thermal conductivity, applied two different covalent functionalization approaches on boron nitride nanotubes (BNNTs) with short polyethylene (PE) chains, and from that work, it can be concluded that with a rather large amount of up to 40% boron nitride nanotubes (BNNTs), a 250% higher thermal conductivity than the pure HDPE matrix can be achieved, which is compared to composites with micro-particles of BN HDPE/EG8/mBN8, while for nano-particles of BN, composites HDPE/EG5/nBN10 or HDPE/EG8/nBN6.5. Furthermore, authors Huang and Qian et al. [36], to improve thermal conductivity, used ultra-high molecular weight polyethylene composites, to which they added a hybrid filler network of boron nitride sheets (BNs) and carbon nanotubes (CNTs) in the matrix. They concluded that it was necessary to add 40% BN sheet and 7 wt.% CNTs to obtain 2.38 W/mK. These results can be compared with the composite HDPE/EG15/mBN15 and HDPE/EG22/nBN6.5, i.e., with a smaller amount of BN in both cases and with the addition of EG, the same value was obtained. The development of the material has reached such a level that the properties of the composite can be significantly improved by adding new additives and with a smaller amount.

## 5. Conclusions

The synergetic effect of both additives EG and BN (micro- and nanoparticles) has a significant impact on the mechanical and thermal properties of polyethylene composite. The addition of PE-g-MAH compatibilizer results in better bonds between the polymer matrix and additives and equal dispersion of particles in the matrix. The differences in the values of tensile strength between the ones of the matrix (25.58 MPa) and the ones with the highest value of additives (24.37 MPa for HDPE/EG15/mBN25 and 24.03 MPa for HDPE/EG15/nBN15) show a negligible drop in strength and a drastic improvement of tensile modulus. At a high percentage of additives, the tensile modulus increased by 105% and 91% for composites with micro- and nano-sized BN particles, respectively.

The impact of particle size is not directly correlated with this characteristic, as the variance in average values is merely 4.5%. The contribution of EG to the increase of tensile strength is dominant compared to BN, which is especially noticeable in the EG/mBN composite group.

Furthermore, the addition of both additives led to the anticipated elevation in the thermal conductivity of the composites. In the case of the HDPE/EG/mBN composite, the thermal conductivity achieved a notable 3.0 W/mK, whereas for the HDPE/EG/nBN composite, the thermal conductivity reached 2.09 W/mK. This outcome provides evidence of established heat transfer pathways at the interface of surfaces. Even at a relatively low ratio of additive (i.e., EG8/mBN8), the thermal conductivity increased by 227% compared to the pure HDPE matrix.

Considering all the findings, composites combining EG and BN could serve as efficient heat conductors in scenarios demanding chemical resistance within the temperature range of 0 to 60 °C (because of the polymer chain degradation at higher temperatures). Further research should prioritize the study of rheological characteristics to determine the optimal parameters for large-scale manufacturing. Based on the optimization outcomes (as presented in Table 18 and Table 20) and the obtained values, the next step involves the fabrication of a pipe to assess the impact of the low strain at yield value on the production process.

## Figures and Tables

**Table 1:** Mass proportion of additives and values of measured properties of HDPE/EG/mBN composite.

Run	HDPE wt.%	Factor A: EG wt.%	Factor B: mBN wt.%	Thermal Conductivity *λ* (W/mK)	Tensile Strength *σ*_m_ (MPa)	Tensile Modulus *E* (MPa)	Strain at Yield *ε*_y_ (%)
1	80	5	15	1.21 ± 0.01	18.84 ± 1.04	1072 ± 98	4.90 ± 1.13
2	70	8	22	1.96 ± 0.14	19.65 ± 0.75	1601 ± 192	2.91 ± 0.40
3	70	15	15	1.96 ± 0.13	23.45 ± 0.48	1723 ± 244	4.94 ± 0.34
4	60	25	15	1.26 ± 0.04	23.85 ± 0.99	1915 ± 324	2.65 ± 0.51
5	20	15	5	1.69 ± 0.10	20.84 ± 1.01	1149 ± 181	5.92 ± 1.2
6	84	8	8	1.21 ± 0.06	19.58 ± 1.95	1164 ± 219	5.71 ± 1.51
7	70	15	15	2.39 ± 0.17	20.13 ± 2.65	1548 ± 152	2.86 ± 0.95
8	60	15	25	0.24 ± 0.00	24.37 ± 3.76	2153 ± 404	2.65 ± 0.28
9	56	22	22	3.01 ± 0.08	23.06 ± 1.77	2087 ± 306	1.85 ± 0.23
10	70	15	15	2.03 ± 0.06	21.86 ± 1.16	1593 ± 154	3.63 ± 0.75
11	70	22	8	1.91 ± 0.07	22.55 ± 1.58	1687 ± 233	3.34 ± 0.4
/	100	/	/	0.37 ± 0.003	25.58 ± 0.99	1104 ± 84	8.52 ± 0.83

The underlined values apply only to the implementation of the analysis of these individual properties, and not for all analyzed properties.

**Table 2:** Mass proportion of additives and values of measured properties of HDPE/EG/nBN composite.

Run	HDPE wt.%	Factor A: EG wt.%	Factor B: nBN wt.%	Thermal Conductivity *λ* (W/mK)	Tensile Strength *σ*_m_ (MPa)	Tensile Modulus *E* (MPa)	Strain at Yield *ε*_y_ (%)
1	64.5	22	13.5	2.79 ± 0.09	23.31 ± 1.85	1899 ± 189	2.32 ± 0.51
2	71.5	22	6.5	2.09 ± 0.07	23.06 ± 1.27	1985 ± 236	3.33 ± 2.99
3	70	15	15	1.95 ± 0.08	24.03 ± 2.86	2209 ± 293	3.66 ± 1.28
4	75	15	10	1.88 ± 0.10	22.80 ± 2.26	1733 ± 101	3.94 ± 0.94
5	75	15	10	1.61 ± 0.07	22.84 ± 2.32	1814 ± 212	4.01 ± 1.26
6	75	15	10	1.44 ± 0.02	21.23 ± 2.07	1477 ± 101	3.86 ± 1.5
7	80	15	5	1.55 ± 0.06	20.83 ± 2.06	1432 ± 227	4.43 ± 4.57
8	85.5	8	6.5	1.11 ± 0.08	20.94 ± 1.17	1328 ± 310	6.61 ± 0.72
9	65	25	10	1.48 ± 0.02	22.99 ± 1.93	2006 ± 241	2.24 ± 0.51
10	85	5	10	0.91 ± 0.04	21.59 ± 1.49	1144 ± 167	6.23 ± 1.23
11	78.5	8	13.5	1.61 ± 0.21	21.95 ± 2.02	1381 ± 168	5.21 ± 1.39
/	100	/	/	0.37 ± 0.003	25.58 ± 0.99	1104 ± 84	8.52 ± 0.83

The underlined values apply only to the implementation of the analysis of these individual properties, and not for all analyzed properties.

**Table 3:** Analysis of variance—influence of EG and mBN on thermal conductivity.

Source	Sum of Squares	df	Mean Square	*F*-Value	*p*-Value (Risk of Rejection of H_0_^1^)
Model	2.26	3	0.7524	17.43	0.0044 significant
A	1.41	1	1.41	32.57	0.0023
B	0.9039	1	0.9039	20.94	0.006
AB	0.032	1	0.032	0.7415	0.4285
Residual	0.2158	5	0.0432		
Lack of Fit	0.1077	3	0.0359	0.6637	0.6476 not significant
Pure Error	0.1081	2	0.0541		
Cor Total	2.47	8			

^1^ H_0_—null hypothesis: there are no factor effects.

**Table 4:** Summary statistics about the model for thermal conductivity of HDPE/EG/mBN composite.

Standard Deviation	0.2077	*R* ^2^	0.9127
Mean	1.93	Adjusted *R*^2^	0.8604
C.V. %	10.76	Predicted *R*^2^	0.683
		Adeq Precision	12.8045

**Table 5:** Analysis of variance—influence of EG and nBN on thermal conductivity.

Source	Sum of Squares	df	Mean Square	*F*-Value	*p*-Value (Risk of Rejection of H_0_)
Model	1.01	2	0.5046	16.82	0.0035 significant
A	0.9653	1	0.9653	32.18	0.0013
B	0.1846	1	0.1846	6.15	0.0477
Residual	0.18	6	0.03		
Lack of Fit	0.0838	4	0.0209	0.4354	0.7833 not significant
Pure Error	0.0962	2	0.0481		
Cor Total	1.19	8			

**Table 6:** Summary statistics for the model for thermal conductivity of HDPE/EG/nBN composite.

Standard Deviation	0.1732	*R* ^2^	0.8486
Mean	1.57	Adjusted *R*^2^	0.7982
C.V. %	11.01	Predicted *R*^2^	0.7249
		Adeq Precision	10.0348

**Table 7:** Analysis of variance—influence of EG and mBN on tensile strength.

Source	Sum of Squares	df	Mean Square	*F*-Value	*p*-Value (Risk of Rejection of H_0_)
Model	26.61	2	13.3	9.78	0.0071 significant
A	22.67	1	22.67	16.67	0.0035
B	3.93	1	3.93	2.89	0.1275
Residual	10.88	8	1.36		
Lack of Fit	5.37	6	0.8945	0.3243	0.8801 not significant
Pure Error	5.52	2	2.76		
Cor Total	37.49	10			

**Table 8:** Summary statistics for the model for tensile strength of HDPE/EG/mBN composite.

Standard Deviation	1.17	*R* ^2^	0.7097
Mean	21.65	Adjusted *R*^2^	0.6371
C.V. %	5.39	Predicted *R*^2^	0.4942
		Adeq Precision	7.8567

**Table 9:** Analysis of variance—influence of EG and nBN on tensile strength.

Source	Sum of Squares	df	Mean Square	*F*-Value	*p*-Value (Risk of Rejection of H_0_)
Model	7.94	2	3.97	9.21	0.0084 significant
A	3.71	1	3.71	8.61	0.0189
B	4.23	1	4.23	9.8	0.014
Residual	3.45	8	0.4314		
Lack of Fit	1.77	6	0.2955	0.3521	0.8644 not significant
Pure Error	1.68	2	0.839		
Cor Total	11.39	10			

**Table 10:** Summary statistics for the model for tensile strength of HDPE/EG/nBN composite.

Standard Deviation	0.6568	*R* ^2^	0.6971
Mean	22.32	Adjusted *R*^2^	0.6214
C.V. %	2.94	Predicted *R*^2^	0.4641
		Adeq Precision	8.1705

**Table 11:** Analysis of variance—influence of EG and mBN on tensile modulus.

Source	Sum of Squares	df	Mean Square	*F*-Value	*p*-Value (Risk of Rejection of H_0_)
Model	1.09 × 10^6^	4	2.734 × 10^5^	48.73	0.0003 significant
A	6.15 × 10^5^	1	6.15 × 10^5^	109.63	0.0001
B	6.427 × 10^5^	1	6.427 × 10^5^	114.57	0.0001
AB	21,640.4	1	21,640.4	3.86	0.1067
A^2^	31,280.84	1	31,280.84	5.58	0.0646
Residual	28,049.55	5	5609.91		
Lack of Fit	11,677.13	3	3892.38	0.4755	0.7314 not significant
Pure Error	16,372.42	2	8186.21		
Cor Total	1.122 × 10^6^	9			

**Table 12:** Summary statistics for the model for tensile modulus of HDPE/EG/mBN composite.

Standard Deviation	74.9	*R* ^2^	0.975
Mean	1652.81	Adjusted *R*^2^	0.955
C.V. %	4.53	Predicted *R*^2^	0.8494
		Adeq Precision	20.1876

**Table 13:** Analysis of variance—influence of EG and nBN on tensile modulus.

Source	Sum of Squares	df	Mean Square	*F*-Value	*p*-Value (Risk of Rejection of H_0_)
Model	7.31 × 10^5^	3	2.437 × 10^5^	14	0.0041 significant
A	7.166 × 10^5^	1	7.166 × 10^5^	41.17	0.0007
B	9491.21	1	9491.21	0.5453	0.4881
AB	4872.04	1	4872.04	0.2799	0.6158
Residual	1.044 × 10^5^	6	17,405.55		
Lack of Fit	42,647.43	4	10,661.86	0.3451	0.8332 not significant
Pure Error	61,785.88	2	30,892.94		
Cor Total	8.354 × 10^5^	9			

**Table 14:** Summary statistics for the model for tensile modulus of HDPE/EG/nBN composite.

Standard Deviation	131.93	*R* ^2^	0.875
Mean	1619.93	Adjusted *R*^2^	0.8125
C.V. %	8.14	Predicted *R*^2^	0.7292
		Adeq Precision	10.1967

**Table 15:** Analysis of variance—influence of EG and mBN on the strain at yield.

Source	Sum of Squares	df	Mean Square	*F*-Value	*p*-Value (Risk of Rejection of H_0_^1^)
Model	15.39	2	7.69	17.08	0.0013 significant
A	5.47	1	5.47	12.14	0.0083
B	9.92	1	9.92	22.02	0.0016
Residual	3.60	8	0.4504		
Lack of Fit	1.39	6	0.2322	0.2101	0.9422 not significant
Pure Error	2.21	2	1.10		
Cor Total	18.99	10			

^1^ H_0_—null hypothesis: there are no factor effects.

**Table 16:** Summary statistics for the model for strain at yield of HDPE/EG/mBN composite.

Standard Deviation	0.6711	*R* ^2^	0.8103
Mean	3.76	Adjusted *R*^2^	0.7628
C.V. %	17.84	Predicted *R*^2^	0.6909
		Adeq Precision	11.0156

**Table 17:** Analysis of variance—influence of EG and nBN on the strain at yield.

Source	Sum of Squares	df	Mean Square	*F*-Value	*p*-Value (Risk of Rejection of H_0_^1^)
Model	13.49	5	2.7	475.96	< 0.0001 significant
A	12.02	1	12.02	2120.07	< 0.0001
B	0.5102	1	0.5102	89.98	0.0007
AB	0.0017	1	0.0017	0.3080	0.6085
A^2^	0.0947	1	0.0947	16.70	0.0150
B^2^	0.0073	1	0.0073	1.29	0.3189
Residual	0.0227	4	0.0057		
Lack of Fit	0.0104	2	0.0052	0.8501	0.5405 not significant
Pure Error	0.0123	2	0.0061		
Cor Total	13.52	9			

^1^ H_0_—null hypothesis: there are no factor effects.

**Table 18:** Summary statistics for the model for strain at yield of HDPE/EG/nBN composite.

Standard Deviation	0.0753	*R* ^2^	0.9983
Mean	3.89	Adjusted *R*^2^	0.9962
C.V. %	1.94	Predicted *R*^2^	0.9907
		Adeq Precision	68.7844

**Table 19:** Constraints and optimization solution for HDPE/EG/mBN composite—version 1.

Name	Goal	Lower Limit	Upper Limit	Solution
Factor A: EG, %	is in range	5	25	5
Factor B: mBN, %	is in range	5	25	20.99
Thermal conductivity, W/mK	max	1.21	3.0	1.9
Tensile strength, N/mm^2^	is in range	18.84	24.37	19.86
Tensile modulus, N/mm^2^	is in range	1072	2153	1458
Strain at yield, %	max	1.85	5.92	3.99

**Table 20:** Constraints and optimization solution for HDPE/EG/mBN composite—version 2.

Name	Goal	Lower Limit	Upper Limit	Solution
Factor A: EG, %	is in range	5	25	17.79
Factor B: mBN, %	is in range	5	25	25
Thermal conductivity, W/mK	max	1.21	3.0	2.52
Tensile strength, N/mm^2^	is in range	18.84	24.37	23.32
Tensile modulus, N/mm^2^	is in range	1072	2153	2119
Strain at yield, %	is in range	1.85	5.92	1.85

**Table 21:** Constraints and optimization solution for HDPE/EG/nBN composite—version 1.

Name	Goal	Lower Limit	Upper Limit	Solution
Factor A: EG, %	is in range	5	22	7.86
Factor B: mBN, %	is in range	5	15	15
Thermal conductivity, W/mK	max	0.91	2.09	1.61
Tensile strength, N/mm^2^	is in range	20.83	24.03	22.67
Tensile modulus, N/mm^2^	is in range	1144	2006	1160
Strain at yield, %	max	2.24	6.23	5.11

**Table 22:** Constraints and optimization solution for HDPE/EG/nBN composite—version 2.

Name	Goal	Lower Limit	Upper Limit	Solution
Factor A: EG, %	is in range	5	22	18.6
Factor B: mBN, %	is in range	5	15	14.14
Thermal conductivity, W/mK	max	0.91	2.09	2.12
Tensile strength, N/mm^2^	is in range	20.83	24.03	23.53
Tensile modulus, N/mm^2^	is in range	1144	2006	1984
Strain at yield, %	is in range	2.24	6.23	2.9

## Data Availability

The data presented in this study are available on request from the corresponding author.
